# Motion-Corrected versus Conventional Diffusion-Weighted Magnetic Resonance Imaging of the Liver Using Non-Rigid Registration

**DOI:** 10.3390/diagnostics13061008

**Published:** 2023-03-07

**Authors:** Je Seung Son, Hee Sun Park, Sungeun Park, Young Jun Kim, Mi Hye Yu, Sung Il Jung, Munyoung Paek, Marcel Dominik Nickel

**Affiliations:** 1 Department of Radiology, Konkuk University Medical Center, 120-1, Neungdong-ro, Gwangjin-gu, Seoul 05030, Republic of Korea; 2 Department of Radiology, Konkuk University School of Medicine, 120-1, Neungdong-ro, Gwangjin-gu, Seoul 05030, Republic of Korea; 3 Department of Diagnostic Imaging, Siemens Healthineers Ltd., The Asset Bldg. 10F, 14 Seocho-Daero 74-gil, Seocho-gu, Seoul 06620, Republic of Korea; 4 MR Application Predevelopment, Siemens Healthcare GmbH, Allee am Roethelheimpark 2, 91052 Erlangen, Germany

**Keywords:** diffusion-weighted imaging, magnetic resonance imaging, motion correction, apparent diffusion coefficient, liver imaging

## Abstract

It is challenging to overcome motion artifacts in diffusion-weighted imaging (DWI) of the abdomen. This study aimed to evaluate the image quality of motion-corrected DWI of the liver using non-rigid registration in comparison with conventional DWI (c-DWI) in patients with liver diseases. Eighty-nine patients who underwent 3-T magnetic resonance imaging (MRI) of the liver were retrospectively included. DWI was performed using c-DWI and non-rigid motion-corrected (moco) DWI was performed in addition to c-DWI. The image quality and conspicuity of hepatic focal lesions were scored using a five-point scale by two radiologists and compared between the two DWI image sets. The apparent diffusion coefficient (ADC) was measured in three regions of the liver parenchyma and in hepatic focal lesions, and compared between the two DWI image sets. Moco-DWI achieved higher scores in image quality compared to c-DWI in terms of liver edge sharpness and hepatic vessel margin delineation. The conspicuity scores of hepatic focal lesions were higher in moco-DWI. The standard deviation values of ADC of the liver parenchyma were lower in the moco-DWI than in the c-DWI. Moco-DWI using non-rigid registration showed improved overall image quality and provided more reliable ADC measurement, with an equivalent scan time, compared with c-DWI.

## 1. Introduction

Diffusion-weighted imaging (DWI) is a technique that explores the random Brownian motion of water molecules in body tissue [1]. DW magnetic resonance imaging (MRI) is a technique that reflects the differences in proton motility between tissues, providing information regarding tissue cellularity and the integrity of cellular membranes [2]. In the field of abdominal imaging, DWI is a promising technique for the detection and characterization of lesions, monitoring of therapeutic responses, and prediction of prognosis in diffuse liver disease [3].

However, the image quality of DWI of the liver is often affected by susceptibility, respiratory motion, cardiac motion, bowel peristalsis, or image distortions [4]. In particular, respiratory motion artifacts are a significant problem in abdominal imaging and may limit the reproducibility of DWI measurements in the liver [5]. Several acquisition methods using different respiration modes have been proposed to address motion artifacts. Breath-hold DWI may improve the detection of small lesions and significantly improve the robustness of DWI data with a short acquisition time [1,6], but some studies indicate that the short imaging duration is at the expense of a decreased signal-to-noise ratio (SNR) and spatial resolution, which may restrict the identification of small lesions [3]. Moreover, only a limited number of b-values can be obtained using breath-holding DWI [5]. Moreover, sufficient breath holding is a challenge for the majority of hospitalized patients. Respiratory-triggered DWI can achieve a high SNR with a sharp margin of the anatomic structures, and it could reduce respiratory motion artifacts compared with free-breathing DWI [4]. However, a long scan time is required to ensure that the necessary repeated averages of the same b-values are acquired at the same respiratory position [3], particularly in patients with irregular breathing patterns, resulting in suboptimal image quality [4]. To reduce the long scan time, a simultaneous multi-slice (SMS) technique based on the simultaneous excitation and acquisition of multiple slices was introduced. It allows for thinner sections and wider coverage, while maintaining image quality, compared with standard single-shot echo-planar DWI [3,7]. However, the SMS technique is also limited in terms of achievable acceleration as the image quality deteriorates at high acceleration factors, particularly through noise amplification [8,9].

Recently, motion correction of abdominal DWI using non-rigid registration was suggested, even though it used respiratory triggering [10]. To correct motion artifacts with higher b-values, motion vectors derived from the non-rigid registration of low b-value diffusion volumes were applied. Preliminary results implied that the proposed method could produce image quality comparable to that of respiratory-triggered DWI while maintaining the reduced scan times.

Therefore, this study aimed to evaluate the image quality of motion-corrected (moco) DWI of the liver using non-rigid registration in comparison to conventional DWI (c-DWI) in patients with liver diseases acquired in the free-breathing condition.

## 2. Materials and Methods

### 2.1. Study Population and Data Collection

This retrospective study was approved by our institutional review board, which waived the requirement for written informed consent owing to its retrospective study design. Between December 2021 and February 2022, 112 adult patients were referred for MRI examination for liver evaluation. Twenty-three patients who underwent hepatic surgery were excluded. Finally, 89 patients (60 men and 29 women; mean age, 63.1 years; age range, 24–87 years) were included in the study. The indications for MRI examination were as follows: follow-up of colorectal malignant tumors with hepatic metastasis (*n* = 36), workup for indeterminate hepatic focal lesions (*n* = 26), follow-up of hepatocellular carcinoma after treatment (trans-arterial chemoembolization and/or radiofrequency ablation) (*n* = 20), workup for elevated tumor markers (*n* = 2), and others (*n* = 5).

### 2.2. Conventional DWI

MRI examinations were performed using a three-Tesla scanner (MAGNETOM Vida, Siemens Healthcare, Erlangen, Germany) with an 18-channel body matrix coil and a 32-channel spine matrix coil. The patients were placed in a head-first supine position. c-DWI images were obtained in the axial plane and free breathing. Acquisition was performed between the early dynamic phase and the hepatobiliary phase of the liver MRI protocol. Trace-weighted diffusion encoding using three orthogonal directions and b-values of 50 s/mm^2^ and 800 s/mm^2^ were employed. Detailed parameters of the c-DWI sequences are listed in Table 1.

### 2.3. Moco-DWI

In addition to the conventional reconstruction, the acquired data were also processed by a scanner-integrated research application that included further steps in the derivation of combined diffusion-weighted images from multiple complex-valued averages with different diffusion weighting, which was developed with a focus on free-breathing acquisitions. As detailed in a previous report [11], the processing first performed an elastic two-dimensional registration of averages with the same b-value and slice position. Furthermore, based on the variations between these averages, the regional signal compensation due to locally corrupted averages was estimated. Averages with the same diffusion weighting and diffusion direction were then adaptively combined as complex-valued images, and trace-weighted magnitude images were derived. In the last step, the latter were aligned again using elastic two-dimensional image registration. For conventional image processing, these were also used to derive apparent diffusion coefficient (ADC) maps.

### 2.4. Image Analysis

#### 2.4.1. Qualitative Analysis

All DWI images were transferred and loaded onto a picture archiving and communication system (Centricity PACS 6.0 SP9, GE Healthcare) for interpretation. Two board-certified radiologists (with 15 and 2 years of experience in abdominal radiology) independently reviewed the DWI images of the two datasets in a random order and evaluated the image quality. The observers were blinded to the patient information, final diagnosis, MRI parameters, and findings of other imaging modalities. Images with b = 800 s/mm^2^ were reviewed. To minimize recall bias, the review sessions for the two DWI sets were separated by at least two weeks. The observers rated the image quality using a five-point Likert scale (5, excellent; 4, good; 3, moderate; 2, fair; or 1, non-diagnostic) with regard to the overall image quality, sharpness of the liver margin and pancreas margin, intrahepatic vessel delineation, cardiac motion artifacts, susceptibility artifacts, motion artifacts derived from respiration or bowel peristalsis, and noise.

#### 2.4.2. Lesion Detection and Characterization

One observer (the observer with more experience) counted all visible focal hepatic lesions showing high signal intensity on DWI images, without referring to other sequences, and rated lesion conspicuity using the following five-point scale: 5, excellent; 4, good; 3, acceptable; 2, poor; 1, undetectable. The size of the lesion and anatomic segment were recorded. In cases of multiple lesions in the liver, the largest lesions were selected. The review sessions of the two image sets had a time gap of at least 2 weeks to minimize recall bias.

#### 2.4.3. Quantitative Analysis

**Figure 1:**
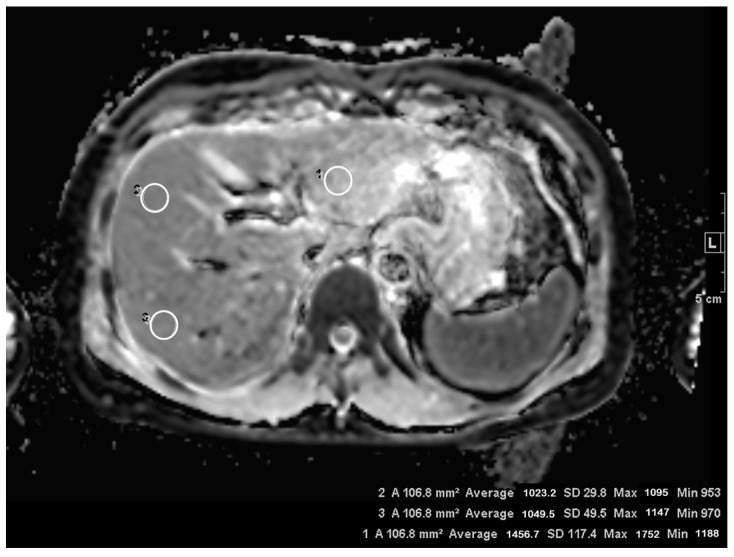
Measurement of apparent diffusion coefficient (ADC) of the hepatic parenchyma on ADC map. Three 1-cm^2^ circular regions of interest were manually drawn at the left lobe (1), right anterior segment (2), and right posterior segment (3) of the liver parenchyma.

A second-year resident in our radiology department measured the ADC values of the liver parenchyma using 1-cm^2^ circular regions of interest (ROIs). Three ROIs were manually drawn at different sites in the liver: the left lobe, right lobe anterior segment, and right posterior segment (Figure 1). ROIs were placed to exclude macroscopic hepatic vessels, the biliary tree, and any focal hepatic lesions, and they were at least 10 mm away from the periphery of the liver [12]. The standard deviation (SD) of the ADC values within each ROI was also recorded. Since one patient was excluded due to an extensive tumor occupying the whole liver parenchyma, 88 patients were included in the measurement of ADC values.

### 2.5. Statistical Analysis

The qualitative scores of c-DWI and moco-DWI were compared using a paired *t*-test. For quantitative analysis, the normality of the data distribution was tested using the Kolmogorov–Smirnov test. Repeated-measures analysis of variance with post hoc analysis or paired *t*-test was used to compare continuous variables. Bonferroni’s post hoc correction was used for multiple comparisons. Interobserver variability for the qualitative analysis was assessed using weighted κ statistics. A κ value < 0.20 indicated poor agreement; 0.21–0.40 indicated fair agreement; 0.41–0.60 indicated moderate agreement; 0.61–0.80 indicated good agreement; and >0.80 indicated excellent agreement. *p*-values < 0.05 were considered statistically significant. Statistical analyses were performed using commercially available software (MedCalc^®^, version 19.5.3; MedCalc Software, Mariakerke, Belgium).

## 3. Results

### 3.1. Subjective Image Quality

**Figure 2:**
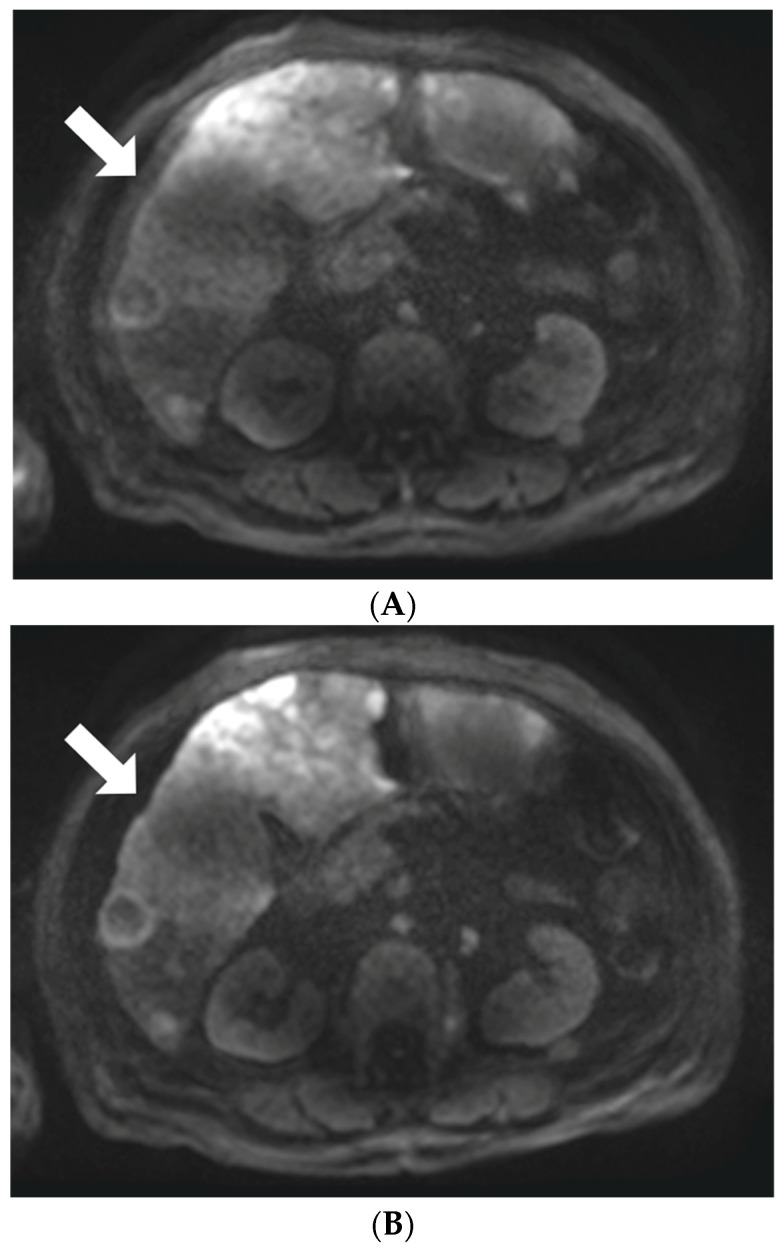
A 72-year-old woman with intrahepatic cholangiocarcinoma who underwent diffusion-weighted imaging (DWI). (**A**) Axial conventional c-DWI (b = 800 s/mm^2^) and (**B**) axial motion-corrected (moco) DWI ((b = 800 s/mm^2^). Margin sharpness of the right liver lobe (arrows) is superior in moco-DWI (**B**) to that in c-DWI (**A**).

**Figure 3:**
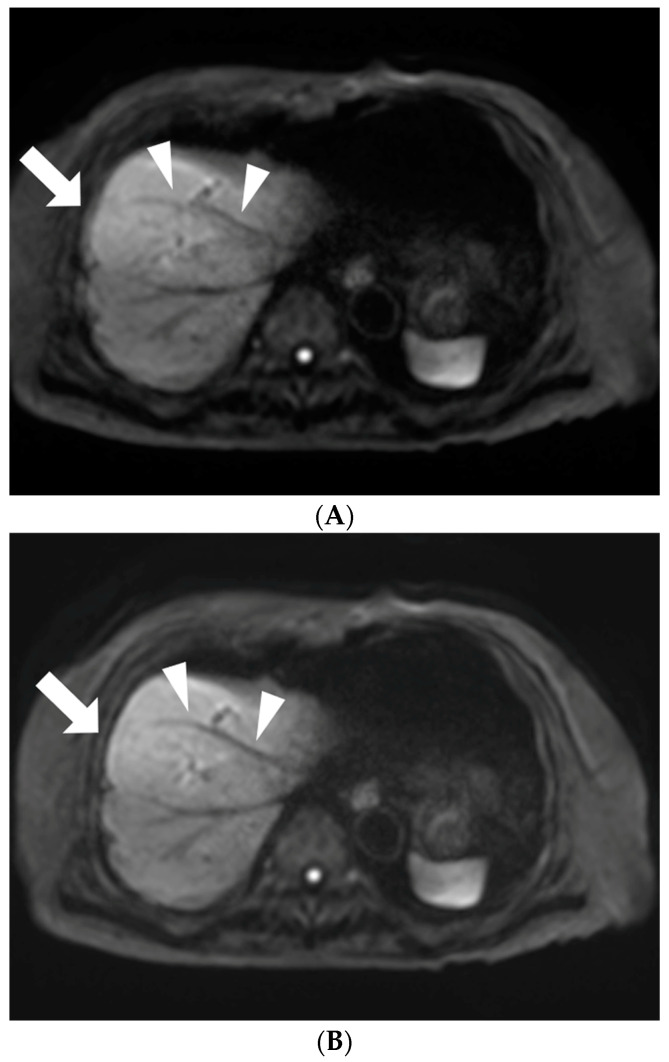
An 81-year-old woman with gallbladder cancer who underwent DWI. (**A**) Axial c-DWI (b = 800 s/mm^2^) and (**B**) axial moco-DWI (b = 800 s/mm^2^). The delineation of the intrahepatic vessel (arrowheads) as well as liver margin sharpness (arrows) are superior in moco-DWI (**B**) to those in c-DWI (**A**).

Moco-DWI achieved significantly higher scores than c-DWI in overall image quality (2.82 ± 0.573 vs. 2.68 ± 0.544, *p* = 0.0018), liver margin sharpness (2.9 ± 0.58 vs. 2.84 ± 0.56, *p* = 0.0244), intrahepatic vessel (2.73 ± 0.633 vs. 2.68 ± 0.668, *p* = 0.0448), and noise (2.49 ± 0.654 vs. 2.43 ± 0.651, *p* = 0.0244). Pancreatic margin sharpness (2.78 ± 0.652 vs. 2.75 ± 0.643), susceptibility artifacts (2.39 ± 0.646 vs. 2.37 ± 0.661), cardiac motion artifacts (2.01 ± 0.581 vs. 1.98 ± 0.547), and motion artifacts (2.38 ± 0.681 vs. 2.35 ± 0.674) were also higher in moco-DWI than in c-DWI, but the difference was not statistically significant (Table 2, Figure 2 and Figure 3). The interobserver agreement for the subjective image quality assessment was moderate to good in both image sets (weighted κ value range 0.495–0.787 for c-DWI, 0.641–0.804 for moco-DWI).

### 3.2. Lesion Visibility and Conspicuity

**Figure 4:**
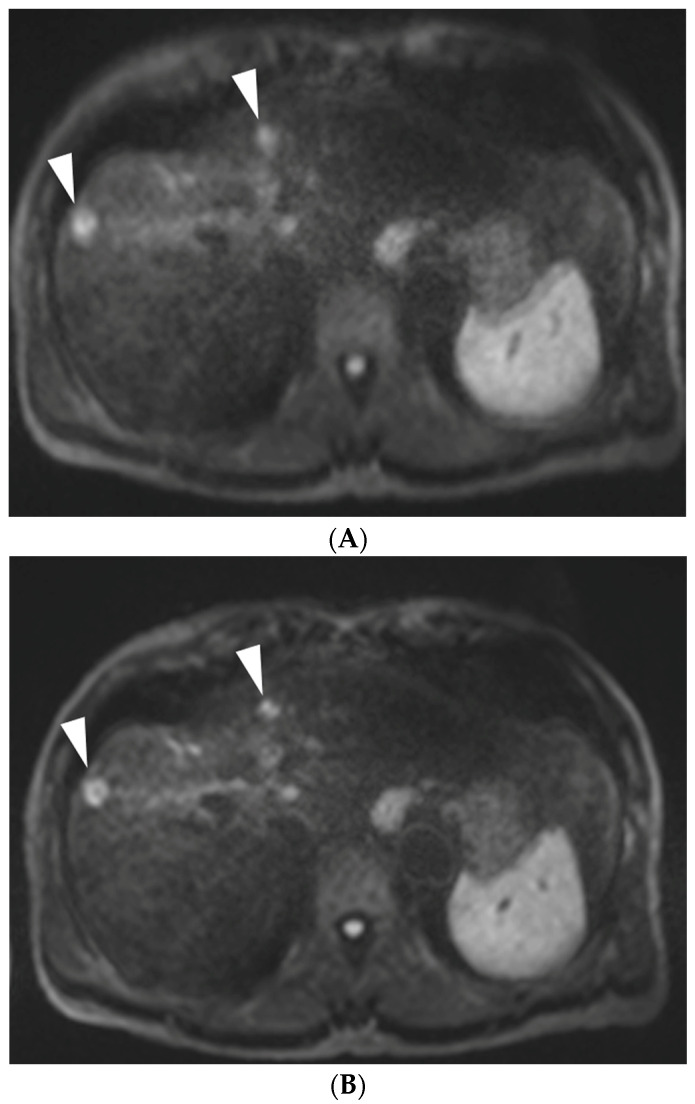
A 57-year-old man with hepatocellular carcinomas who underwent DWI. (**A**) Axial c-DWI (b = 800 s/mm^2^) and (**B**) axial moco-DWI ((b = 800 s/mm^2^). The conspicuity of the two hepatocellular carcinoma nodules (arrowheads) is better in moco-DWI (**B**) than in c-DWI (**A**).

Sixty-eight focal lesions in 47 patients were detected on both c-DWI and moco-DWI images (mean lesion diameter 2.35 cm, diameter range, 0.2 to 9.5 cm). The conspicuity score of the lesions was higher on moco-DWI than on c-DWI, but the difference was not significant (3.27 ± 1.02 vs. 3.25 ± 1.04). An example is shown in Figure 4.

### 3.3. ADC Measurement

On both DWI images, ADC values were significantly higher in the left liver lobe than in the right anterior segment or the right posterior segment (*p* < 0.001 in c-DWI and moco-DWI), whereas the ADC values of the right anterior segment and those of the right posterior segment were not significantly different (Table 3). When a pairwise comparison was performed of the ADC values between the two DWI image sets at different liver locations, those in moco-DWI showed overall lower values than those in c-DWI, but the difference was not statistically significant at any location in the liver.

As for the SD of the ADC within each ROI, the SD was significantly higher in the left liver lobe than in the right anterior or right posterior segment of the liver in both DWI image sets (*p* < 0.001 in c-DWI and moco-DWI). Moco-DWI showed overall lower SD values than c-DWI in all measured liver locations. However, the difference was not statistically significant (Table 4).

## 4. Discussion

Our study results demonstrated that the image quality was significantly improved in moco-DWI images compared to c-DWI images. Using the moco-DWI method, scores for overall image quality, liver margin sharpness, intrahepatic vessel delineation, and noise were significantly higher than those in the c-DWI images. This result is in accordance with that of a preliminary study that investigated SMS-accelerated DWI with motion correction [3]. In the mentioned study, moco-DWI images showed superior sharpness of the liver edge and vessel contour and overall image quality compared to those of c-DWI images. DWI in abdominal imaging is obtained by averaging single-shot image acquisitions, and averaging results in blurred images by motion [3].

However, even though artifacts associated with cardiac motion, susceptibility, and the sharpness of the pancreatic margin were slightly improved after applying the moco-DWI technique, the difference was not statistically significant compared to c-DWI. Cardiac motion during the systolic phase causes distortion of the left hepatic lobe, which is located immediately below the heart [13]. Consequently, this results in regional deformation, leading to signal loss in DWI and artificially increased ADC values. Moreover, the image quality of DWI of the left lobe of the liver and pancreas is frequently impaired due to susceptibility artifacts caused by the stomach and bowel gas [4]. Compared with the right lobe of the liver, the left lobe is more vulnerable to artifacts owing to the closer cardiac abutment and stomach gas. To overcome cardiac pulsation artifacts, further investigation using cardiac-gated or navigator echo-correction methods is necessary. In a recent study of DWI using deep learning reconstruction, the margin sharpness of the pancreas and the left lobe of the liver was significantly improved compared to c-DWI [4]. The presumed reason is the decreased susceptibility artifacts related to stomach and bowel gas through the increased parallel imaging factor and the consequent corresponding shortening of the echo train lengths [4]. Along with the motion correction technique used in our study, deep learning reconstruction may be a promising method for correcting various motion artifacts in DWI.

In the quantitative analysis, ADC values were higher in the left lobe of the liver than in the right lobe, either anterior or posterior. This result is in agreement with previous studies and the results of the qualitative analysis in our study [14,15,16,17]. As mentioned earlier, the higher ADC values in the left lobe of the liver can be explained by signal losses caused by cardiac motion [6,18,19]. According to a study investigating the effect of cardiac motion on DWI, the signal intensities of the right and left liver lobes are also affected by cardiac motion [13]. A possible explanation for the signal loss of the right liver lobe is the direct impact of the heart on the liver and immediate post-systole arterial pulsation, leading to the propagation of a compression wave, causing non-rigid body motion of the right liver lobe [13]. Therefore, signal loss in the right liver lobe is considerable in DWI, but a larger volume and the low effect of stomach gas might have accounted for the relatively low signal loss in the right liver lobe than in the left lobe.

The overall SD of the ADC values was significantly higher in the left liver lobe than in either the right anterior segment or the right posterior segment of the liver in both DWI image sets. These results seem to be in line with the results that the signal intensities were significantly higher in the left liver lobe than in the right anterior or posterior segment of the liver, because both results reflect that the left liver lobe is more vulnerable to artifacts, probably caused by cardiac motion. Furthermore, when a pairwise comparison of the SD between c-DWI and moco-DWI was performed, the overall SD was slightly lower in moco-DWI in all three locations in the liver, although the differences were not statistically significant. The lower SD in moco-DWI than that in c-DWI suggests that it is more homogeneously distributed, less scattered, and, therefore, more reliable [20,21,22]. Similarly, the SNR was significantly higher in moco-DWI, and the ADC values at multiple sites in the liver were significantly lower than those in c-DWI in a study using SMS-DWI [3]. In several reports, lower SDs in ADC measurements were achieved with superior DWI protocols, including DWI with deep learning reconstruction [4].

In our study, DWI images were obtained using the free-breathing method. In addition to the liver’s anatomic location, imaging techniques also affect ADC measurement [15,16,17]. In addition to the cardiac motion addressed above, respiratory motion artifacts are also one of the most important factors [6,21]. Four techniques are widely used in clinical practice to compensate for respiratory motion factors: breath-holding, free-breathing, respiratory-triggered, and navigator-triggered DWI. In a prospective study comparing the reproducibility of ADC measurements in normal liver parenchyma using the four different respiration control techniques, DWI using the free-breathing technique showed better reproducibility and a shorter acquisition time than DWI using the other three techniques [14]. A possible explanation for this result is that more excitations generate more reproducible ADCs. More excitations can improve DWI acquisition during the diastolic phase, resulting in a higher signal than that in the systolic phase [13]. Further, the signal loss is compensated for by multiple signal acquisitions. Another study with a similar design suggested that the reproducibility of ADC measurement of the liver parenchyma was comparable in breath-hold and free-breathing DWI techniques compared with respiratory-triggered DWI [23]. Since these studies were conducted in healthy volunteers, further study in a heterogeneous patient population is necessary, and application of the optimal motion correction method in the free-breathing DWI in our study may be promising in terms of the reproducibility of ADC measurement.

This study has several limitations and future directions to be addressed. First, although the study revealed the superior overall image quality of moco-DWI to c-DWI with improved liver margin sharpness and intrahepatic vessel delineation, we failed to determine a statistically significant improvement in the results of moco-DWI regarding cardiac motion and susceptibility artifacts. Because image degradation caused by cardiac motion and susceptibility artifacts may be fatal in the interpretation of DWI images and the measurement of ADC, further studies should be conducted to clarify the limitations of the current motion correction technique. Second, although the overall image quality was improved, it is not clear whether the diagnostic capacity of moco-DWI is superior to that of c-DWI, because solely better image quality does not affect the patient’s treatment or the diagnostic efficacy. However, we believe that the improved conspicuity of the focal liver lesion in moco-DWI would contribute to improved confidence in or detection of smaller lesions, and therefore overall improved diagnostic performance in following studies. Third, even though the image quality using moco-DWI with the free-breathing technique is improved compared to that using c-DWI, it is still not comparable to breath-hold or respiratory-triggered DWI. Therefore, further study comparing moco-DWI using different breathing techniques would be necessary. Fourth, the comparison of ADC measurements between the hepatic focal lesions in the two DWI image sets was not performed because we simply tried to assess the visibility of high-signal-intensity lesions on DWI images, and only the comparison of the subjective assessment of lesion detection and lesion conspicuity was performed. From the perspective of clinical practice, the evaluation of focal liver lesions may be more relevant. Fifth, since DWI using deep learning reconstruction exhibited promising results in another study [4], applying our motion correction method to deep learning DWI may be interesting and should be considered as a future research topic.

## 5. Conclusions

In conclusion, moco-DWI using non-rigid registration showed improved overall image quality and provided more reliable ADC measurements, with an equivalent scan time, compared to the corresponding parameters associated with c-DWI.

## Figures and Tables

**Table 1:** Diffusion-weighted imaging parameters.

Protocol Parameter	
b-values (s/mm^2^)	50, 800
TR/TE (ms)	5200/51
Field-of-view (mm^2^)	379 × 260
Matrix	140 × 96
In-plane resolution (mm^2^)	1.36 × 1.36 (interpolated)
Slice thickness (mm)	5
Number of slices	35
Flip angle (degree)	90
Parallel imaging factor	2
Number of excitations	2 (b = 50 s/mm^2^), 4 (b = 800 s/mm^2^)
Fat suppression	SPAIR
Acquisition time (m:s)	03:36 (free breathing)

TR: repetition time, TE: echo time, SPAIR: spectral attenuated inversion recovery.

**Table 2:** Comparison of qualitative analysis between c-DWI and moco-DWI.

Subjective Image Analysis	c-DWI	moco-DWI	*p*-Value
Overall image quality	2.68 ± 0.544	2.82 ± 0.573	0.0018
Liver margin sharpness	2.84 ± 0.56	2.9 ± 0.58	0.0244
Intrahepatic vessel delineation	2.68 ± 0.668	2.73 ± 0.633	0.0448
Pancreas margin sharpness	2.75 ± 0.643	2.78 ± 0.652	0.1586
Susceptibility artifact	2.37 ± 0.661	2.39 ± 0.646	0.1586
Cardiac motion artifact	1.98 ± 0.547	2.01 ± 0.581	0.0832
Motion artifact	2.35 ± 0.674	2.38 ± 0.681	0.0832
Noise	2.43 ± 0.651	2.49 ± 0.654	0.0244

Data are mean ± standard deviation, and scores are averages of the two observers. c-DWI: conventional diffusion weighted imaging, moco-DWI: motion-corrected diffusion weighted imaging.

**Table 3:** Comparison of ADC measurement in liver parenchyma between c-DWI and moco-DWI.

					Post Hoc Analysis		
	Left	Right Anterior	Right Posterior	*p*-Value	Left vs. Right Anterior	Left vs. Right Posterior	Right Anterior vs. Right Posterior
c-DWI	1.194 ± 0.028	1.030 ± 0.149	1.023 ± 0.02	<0.001	<0.001	<0.001	1
moco-DWI	1.183 ± 0.027	1.027 ± 0.014	1.013 ± 0.02	<0.001	<0.001	<0.001	1
*p*-Value	0.0853	0.4468	0.0584				

Data are mean ± standard deviation. ADC: apparent diffusion coefficient.

**Table 4:** Comparison of SD of ADC measurement in liver parenchyma between c-DWI and moco-DWI.

					Post Hoc Analysis		
	Left	Right Anterior	Right Posterior	*p*-Value	Left vs. Right Anterior	Left vs. Right Posterior	Right Anterior vs. Right Posterior
c-DWI	0.096 ± 0.005	0.078 ± 0.005	0.072 ± 0.004	<0.001	0.0031	<0.0001	0.3096
moco-DWI	0.092 ± 0.005	0.076 ± 0.004	0.07 ± 0.004	<0.001	0.0281	0.0001	0.1535
*p*-Value	0.2866	0.3328	0.4789				

Data are mean ± standard deviation. SD: standard deviation; ADC: apparent diffusion coefficient.

## Data Availability

Not available.
